# An evaluation of repellency and feeding inhibition of ethno-medicinal plants against major malaria vectors in southern Ethiopia

**DOI:** 10.1186/s13071-021-04694-6

**Published:** 2021-04-07

**Authors:** Tekle Olbamo, Endashaw Esayas, Tizazu Gebre, Fekadu Massebo

**Affiliations:** 1 Department of Animal Science, Jinka University, Jinka, Ethiopia; 2 Department of Biology, Arba Minch University, Arba Minch, Ethiopia; 3 Malaria and Neglected Tropical Diseases Research Directorate, Armauer Hansen Research Institute, Addis Ababa, Ethiopia

**Keywords:** Ethno-medicinal plant, Feeding inhibition, Indoor density, Kolla Shara, Malaria vector, Repellency, Smoking

## Abstract

**Background:**

Plant-based mosquito control methods may use as a supplementary malaria vector control strategy. This study aimed to evaluate the effect of smoking ethno-medicinal plants on indoor density and feeding activity of malaria vectors at early hours of the night and its residual effect after midnight in southern Ethiopia.

**Methods:**

Both field and tent trials were conducted to evaluate the impact of smoking *Juniperus procera* leaves*, Eucalyptus globulus* seeds and *Olea europaea* leaves in Kolla Shara Village from July 2016 to February 2017. For the field trial, five grass-thatched traditional huts (three for ethno-medicinal plants and two as control [only charcoal smoking and non-charcoal smoking]) were used. Indoor host-seeking mosquitoes were collected by CDC light traps. A Latin square design was employed to minimize the bias due to the variation in house location and different sampling nights. For the tent experiment, 25 3–5-day-old starved wild female *Anopheles* mosquitoes reared from the larvae were released into the tents where a calf was tethered at the mid-point of each tent.

**Results:**

A total of 614 *Anopheles* mosquitoes belonging to 5 species were collected from 5 huts, of which 93.4% was *An. arabiensis*; *O. europaea, E. globulus* and *J. procera* reduced the indoor density of *An. arabiensis*, with the mean percentage drop of 80%, 73% and 70%, respectively. In the tent trial, smoking of these plants had significant knockdown effects and inhibited feeding on the calves (*F* = 383.5, DF = 3, *P* < 0.01). The mean knockdown effect due to *O. europaea* was relatively high (17.7 ± 0.54; 95% CI 16.8–18.6), while it was only 0.9 ± 0.1 (95% CI 0.29–1.52) in the control tents. All the test plants used in the tent trial caused significantly inhibited feeding activity of *An. arabiensis* on the host (*F* = 383.5, DF = 3, *P* < 0.01). About 94.5%, 89.5% and 86% of mosquitoes were unfed because of the smoking effect of *O. europaea*, *E. globulus* and *J. procera*, respectively, whereas only 19.5% were unfed in the control tent.

**Conclusions:**

Smoking ethno-medicinal plant materials reduced indoor density of malaria vectors and inhibited feeding on calves inside the tents. Thus, plant-based mosquito control methods may play a vital role in reducing mosquito bites in the early hours of the night and thereby reduce residual malaria transmission.

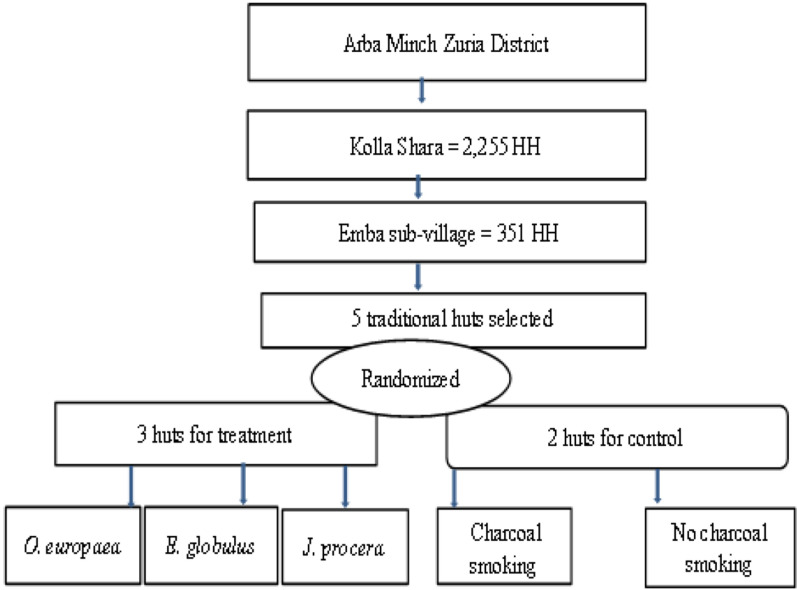

## Background

Malaria claims millions of lives globally, particularly in malaria-endemic countries. The burden of the disease continues to cause a considerable amount of morbidity and mortality in sub-Saharan Africa [1]. In 2018, 93% of malaria cases and 94% of malaria-related deaths were from sub-Saharan African countries [2]. In Ethiopia, malaria is the leading vector-borne disease, and the population living in malaria risk areas is still suffering from it [3–5].

Vector control is a key intervention for the interruption of malaria transmission and elimination efforts. Currently, the main malaria vector control interventions are indoor residual spraying (IRS) and long-lasting insecticidal nets (LLINs) [2]. The changing behavior of the vectors, such as early hours and outdoor biting, as well as resting, could maintain malaria transmission even in areas with maximum coverage of the existing tools [6]. This shifting in resting and feeding behaviors in the main malaria vectors in response to control methods might have a negative impact on the current indoor-based malaria vector control interventions [7, 8]. The time of feeding in both endophagic and exophagic vector populations may also be of critical importance if it occurs in the hours outside of LLIN use. Some people may get infectious bites before they finish their outdoor activities and are confined to the bednets. For instance, the principal malaria vector, *Anopheles arabiensis*, in the northern and south-central parts of Ethiopia shows exophilic behavior that might challenge IRS-based malaria control interventions. Thus, heterogeneity in mosquito biting and resting behavior suggests there is still a need to design additional tools that target the vectors not addressed by the current malaria interventions [6–8]. Furthermore, *An. arabiensis* has developed resistance to most public health insecticides, which has increased interest in searching for new insecticides and alternative vector control approaches [9, 10].

In Ethiopia, people in malaria-endemic villages use local ethno-medicinal plants to repel mosquitoes and other nuisance insects. Among them, smoking plants to drive away mosquitoes and other insects is a common practice. Some plants have repellent properties that could be a part of vector control interventions [11, 12]. Kolla Shara village is one of the malaria-endemic villages in the Arba Minch Zuria district where IRS and LLINs are the two main malaria intervention tools. *An. arabiensis* is also the principal malaria vector in the village [13]. According to the unpublished data of Arba Minch Zuria District Health Office 2016, despite high coverage of IRS and LLINs, malaria continued to be a major public health problem in the village. Hence, it is clear that the existing malaria control interventions are insufficient to stop early hour indoor residual transmission, and there is a need for supplementary interventions to control malaria. Therefore, this study aimed to evaluate the effect of smoking ethno-medicinal plants on indoor density and feeding activity of malaria vectors in the early hours of the night and the residual effect after midnight in southern Ethiopia.

## Methods

### Study area description

**Fig. 1:**
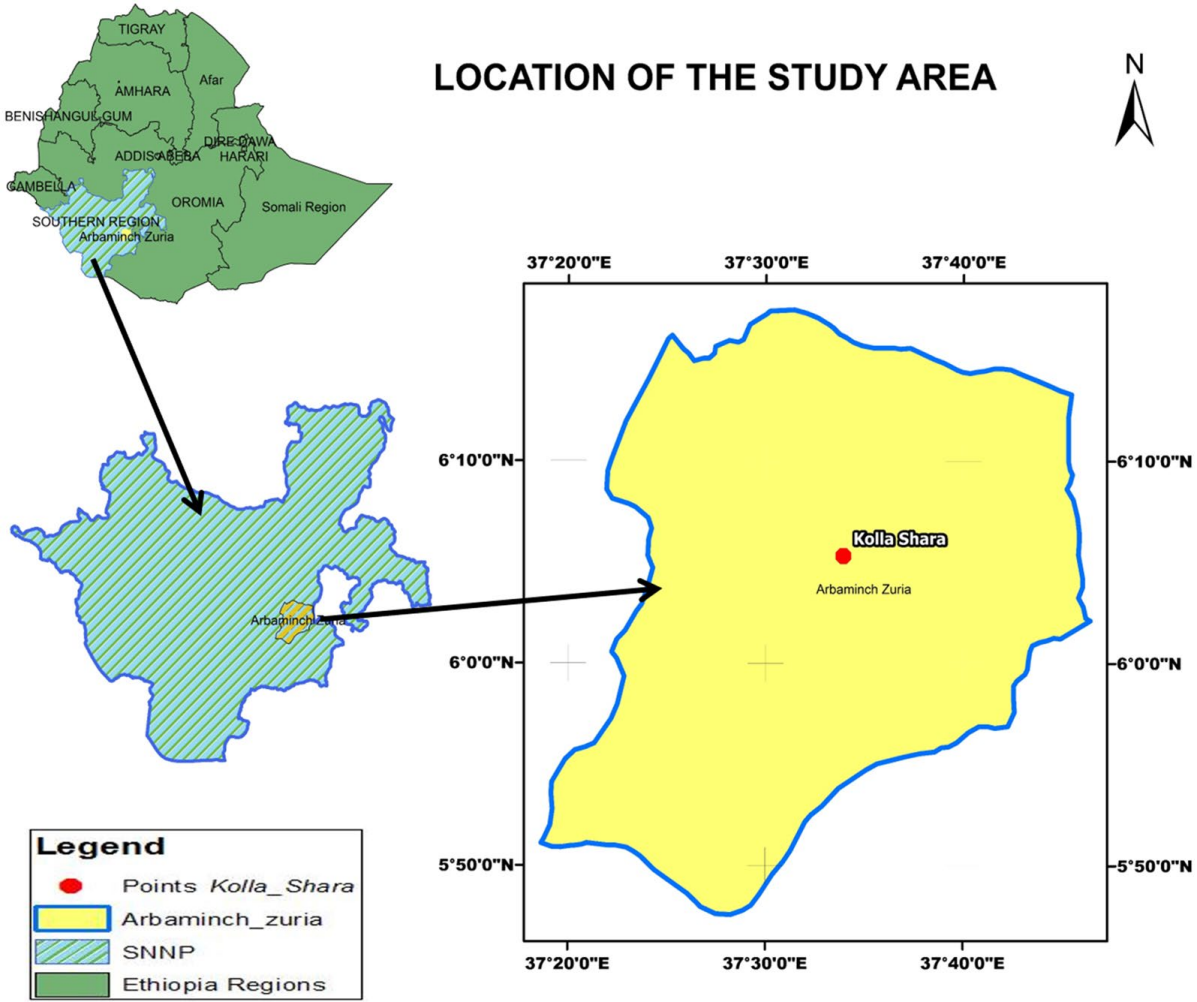
Map of Kolla Shara village in Arba Minch Zuria district, southern Ethiopia

The field trial was carried out in Kolla Shara village situated 490 km southwest of Addis Ababa, the capital city of Ethiopia. The village is located 6 km north of Arba Minch town (Fig. 1). The mean annual temperature of the village ranges from 18 to 35 °C, and the mean annual rainfall ranges from 750 to 1060 mm. The total number of households in the village is 2255 with a total population of about 10,060 (Arba Minch Zuria District Agricultural Office Unpublished Data, 2015). The village has one government health post located at 6°05′N, 37°34′E and the average altitude of the village is 1390 m above sea level. The village was selected purposely for this study because of its malaria endemicity, and there are many irrigation canals within the village that may serve as potential breeding sites for mosquitoes. Moreover, the Hare River, which originates from the highland, crosses the village near the eastern border and flows southeast to join to Lake Abaya. The river has high potential for irrigation and forms small pockets of water in dry seasons that are ideal mosquito breeding sites. *An. arabiensis* is the most abundant species in the village followed by *An. pharoensis*. The other anopheline species (*An. tenebrosus, An. funestus* group, *An. ziemanni, An. demeilloni* and *An. pretoriensis*) are found in very small proportions [13]. The typical rural houses in the village are traditional circular huts constructed from wood, grass and mud.

### Description of the test plants

Three medicinal plants with traditional insect-repellent properties were selected based on bio-ethnological knowledge of the farmers and local residents and literature information [14]. The leaves of *Juniperus procera* Hochst. Ex Endl (local name: *Tid*), fruits of *Eucalyptus globulus* Labill (local name: *Nech Bahir Zaf*) and leaves of *Olea europaea* (local name: *Woira*) were used for the test. These plants are widely used by community members to deter house-entering insects. Ethno-medicinal plant materials were collected from Kolla Shara village (*O. europaea*) and Dega Ocholo village (*E. globulus* and *J. procera*).

### Field trial procedures

**Fig. 2:**
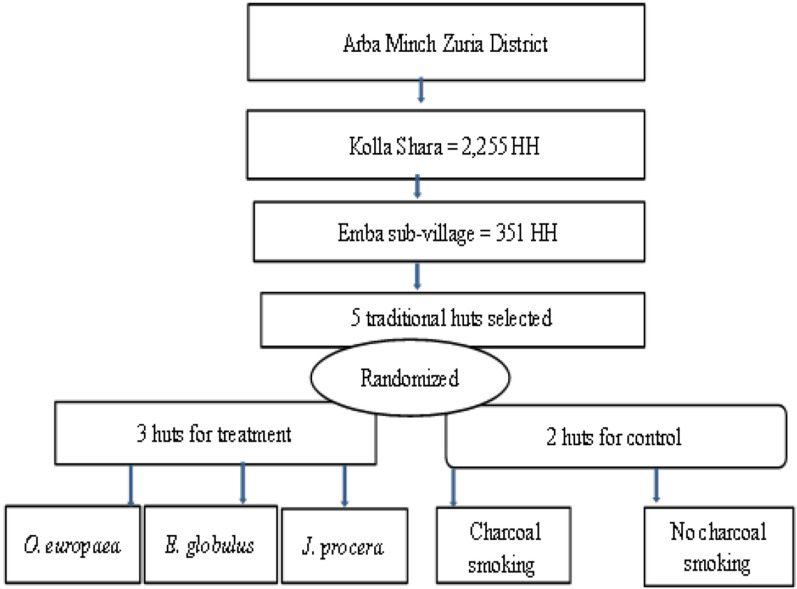
Flow chart of the field study design

Five traditional huts with mud walls, grass thatched roofs and unscreened windows and some openings on the eaves and doors were selected. The huts were similar in type to minimize the variation and to use the same amount of plant materials for smoking. In order to avoid the diversion effect of the smoke to the next huts (in both the control and treatment groups), the distance between huts was approximately 100 to 200 m from each other. Three huts were used for smoking ethno-medicinal plant materials (treatment huts), and two huts were used as control. The huts for the experimental trial (treatments) were assigned randomly to *J. procera, E. globulus*, *O. europaea* and control (only charcoal smoking and non-charcoal smoking) on the first sampling night and then rotated after each trial to minimize the bias due to spatial and temporal variation of mosquito density in different houses (Fig. 2). A traditional clay stove was used for smoking in both the treatment and control huts. A Latin square design (5 × 5) was applied to measure the variation in mosquito catches between intervention and control huts. The residual effect of smoking was evaluated by comparing between control and treatment groups using the number of mosquitoes collected after midnight (smokeless hours).

In each hut, 100 g of test ethno-medicinal plants was placed directly on the traditional clay stove above burned charcoal at the beginning of each trial. Every hour, an additional 30 g of plant material was added to the clay stove for smoking in each hut until midnight (18:30–24:00). Then, the charcoal was removed from the clay stove in both the treatment and control huts at midnight when people slept. Moreover, 300 g of charcoal was added at the beginning followed by an additional 150 g of charcoal every hour in both the treatment and control huts and then removed at midnight. The clay stove with smoking plant material was put on the ground at the mid-point to have uniform distribution of smoke inside the huts. The experiment was conducted three times a week with a 2-day gap to avoid a residual effect of the previous night's smoke. The trial for each test ethno-medicinal plant was repeated seven times in each hut and conducted for 35 nights in a 6-month period.

### Entomological sampling and identification of species

Indoor host-seeking mosquitoes were collected using Centers for Disease Control and Prevention (CDC) light traps in both treatment and control huts. CDC light traps were installed on a wall or roof at the foot end of the bed where the human occupant was protected by untreated bed nets [15]. Other occupants of the houses were left to use the bed nets provided by the district health office as part of the routine malaria control program. Traps were operated between 18:30 and 6:00 (including smoking and smokeless hours). All the CDC light traps containing mosquitoes were removed at 24:00 in each hut and replaced by new traps to determine the residual effect of smoking plants. The replaced traps were then removed at 6:00 the next morning. After each night of collection, CDC light traps were washed thoroughly with detergent, soap and water to avoid the residual effect of treatment on the next sampling night. Live female *Anopheles* mosquitoes were killed by freezing, and identification was done using morphological keys [16]. The abdominal status was determined as unfed, freshly fed, half-gravid and gravid under the light microscope. The specimens were stored separately in Eppendorf tubes containing silica gel as the drying agent.

### Tent experimental design

#### Rearing *Anopheles* larvae and pupae

*Anopheles* larvae and pupae were collected along the shore of the Kulfo River using a dipper. After collection, the mosquito larvae and pupae were transported in well-labeled plastic bottles to Arba Minch University medical entomology laboratory and maintained at 26 ± 2 °C and 65 ± 5% relative humidity. The pupae were transferred into the glass beaker and put in labeled cages for adult emergence and were provided with sterilized 10% sugar solution. The emerged adults were identified by using a morphological identification key [16]. Only *An. gambiae* complex (*An. arabiensis*) was used for the experiment.

#### Feeding inhibition experiment

Five healthy calves (zebu breed) aged 2–3 years old were rented from the community for the feeding experiment. The cattle were treated with prophylactic drug for 15 days before commencing the trial to avoid stress and other health-related problem. The owners took care of the cattle to prevent unnecessary stress. A release-recapture trial was carried out in the tents. Five white tents with dimensions of 2 m × 2 m × 2 m were deployed 20 m from each other to avoid the effect of plant smoke on the next tent. The tents have a zipper which is used to open and close the tent while releasing and collecting the mosquitoes. At the mid-point of each tent, a fixed object (crush) was constructed to tether and fasten the animal, limiting its range of movement and disturbance during the trial. A white sheet (2.5 m × 2.5 m) was spread on the ground for easy recognition of knocked-down mosquitoes. The three cattle were randomly assigned for treatment while the other two cattle were used as the control and then rotated. After each calf was introduced into each tent, a clay stove with a smoking local ethno-medicinal plant was placed at the corridor near the tent zipper; then, 20 3–5-day-old starved wild adult *Anopheles* mosquitoes were released into each tent by opening the zipper at 18:30. The gap was closed to prevent mosquitoes from exiting the tent. At the beginning of the trial, 300 g charcoal and 100 g of test plant material were used. To produce smoke, the plant material was placed directly on traditional a clay stove with burned charcoal. The tents (control with charcoal only and treatment with plant material and charcoal) were smoked until midnight (24:00) by adding 30 g plant material and 150 g of charcoal every hour.

At midnight, the knockdown mosquitoes were collected from the ground, and the live mosquitoes were aspirated and killed immediately by chloroform to avoid blood digestion and kept separately in the paper cup. The abdominal stage of mosquitoes was determined under the light microscope. The trial was conducted for a total of 15 nights, four times a week for 2 months. The trial for each test plant material was repeated three times in each tent. After each trial, the tents were cleaned and stay opened for 1 day to avoid a residual effect of smoke from the previous night.

#### Outcome variables

The first primary outcome variable was the indoor density of malaria vectors. The second primary outcome variable was the number of unfed malaria vectors due to the smoking in the tent experiment. The secondary outcome variable was the species composition of malaria vectors in the study area.

### Statistical analysis

Data were first entered into Excel and then imported into IBM^®^ SPSS^®^ (version 20, IBM Corp, Armonk, NY, USA) for analysis. A generalized linear model was used to determine the significant difference in mean density of mosquitoes in treatment and control huts. Analysis of variance was used to measure the mean percentage protection, knockdown effect and feeding inhibition of smoking test plants. Pearson’s chi-square test was used to describe the associations of variables. Test of significance was estimated assuming α at 0.05, and a *P* value < 0.05 was considered significant. Comparison was made between control and treatment groups as well as between treatment groups.

## Results

### Anopheles species composition

A total of 614 adult female *Anopheles* mosquitoes belonging to five species, *Anopheles arabiensis, An. demeilloni, An. funestus* group*, An. pretoriensis* and *An. pharoensis*, were collected from five huts selected for the field experiment trial. *An. arabiensis* was the predominant (93.4%; 574/614) species. The other four species, *An. demeilloni* (2.3%; 14/614), *An. funestus* group (2%; 12/614), *An. pretoriensis* (1.6%; 10/614) and *An. pharoensis* (0.7%; 4/614), were found in small numbers.

### Overall effect of smoking on indoor density of malaria vectors

The evaluation of indoor density of host-seeking malaria vectors after smoking medicinal plants shows there was a significant difference between the test plants and charcoal control (*F* = 33.7, DF = 4, *P* < 0.001). However, there was no significant difference among the test plants (*P* = 0.32) or between charcoal and not charcoal smoking (*P* = 31) (Table 1).

**Table 1:** The overall effect of smoking medicinal plants on indoor density of host-seeking *Anopheles* mosquitoes

Tests	Number collected	Mean (95% Wald CI)	Mean ratio (95% CI)	% Reduction	*P*-value
Non-charcoal smoking	236	6.6 (5.4–8.2)	–	–	–
Charcoal smoking	213	5.9 (4.9–7.4)	1	–	–
*J. procera*	66	1.8 (1.4–2.4)	0.3 (0.12–0.65)	70	< 0.001
*E. globulus*	57	1.6 (1.2–2.2)	0.27 (0.04–0.65)	73	< 0.001
*O. europaea*	42	1.2 (0.8–1.7)	0.2 (0.04–0.65)	80	< 0.001

### Effect of smoke on indoor density of mosquitoes during smoking hours

Smoking *O. europaea, E. globulus* and *J. procera* had a significant effect on the indoor density of host-seeking malaria vectors (*F* = 43.3, DF = 4; *P *< 0.001). *Olea europaea* induced the highest reduction (85%), followed by *J. procera* (74%) and *E. globulus* (71%) (Table 2).

**Table 2:** The effect of smoking medicinal plants on indoor density of host-seeking *Anopheles* mosquitoes during smoking hours

Tests	Number collected	Mean (Wald 95% CI)	Mean ratio (95% CI)	% Reduction	*P*-value
Non-charcoal smoking	144	4.0 (3.4-4.9)	–	–	–
Charcoal burning	135	3.7 (3.2–4.4)	1	–	–
*J. procera*	33	0.9 (0.7–1.3)	0.26 (0.07–0.24)	74	< 0.001
*E. globulus*	41	1.1 (0.8–1.6)	0.29 (0.21–0.64)	71	< 0.001
*O. europaea*	21	0.58 (0.4–0.9)	0.15 (0.07–0.15)	85	< 0.001

### Residual effect of smoking on indoor density of malaria vectors

*Olea europaea, E. globulus* and *J. procera* significantly reduced the indoor density of host-seeking mosquitoes after midnight because of the residual effects inside the treatment huts compared to control huts (*F* = 12.4, DF = 4; *P *< 0.001). The mean percentage reduction was 81%, 74% and 61% for *E. globulus*, *O. europaea* and *J. procera,* respectively (Table 3).

**Table 3:** Residual effect of smoking medicinal plants after midnight on indoor density of host-seeking *Anopheles* mosquitoes

Tests	Number collected	Mean (95% Wald CI)	Mean ratio (95% CI)	% Reduction	*P*-value
Non-charcoal smoking	91	2.5 (1.9–3.7)	–	–	–
Charcoal smoking	81	2.3 (1.7–3.3)	1	–	–
*J. procera*	33	0.9 (0.7–1.3)	0.39 (0.01–0.81)	61	< 0.001
*E. globulus*	16	0.44 (0.3–0.8)	0.19 (0.01–0.81)	81	< 0.001
*O. europaea*	21	0.6 (0.4–0.9)	0.26 (0.01–0.81)	74	< 0.001

### Knockdown effect of smoking plants in the tent experiment

**Fig. 3:**
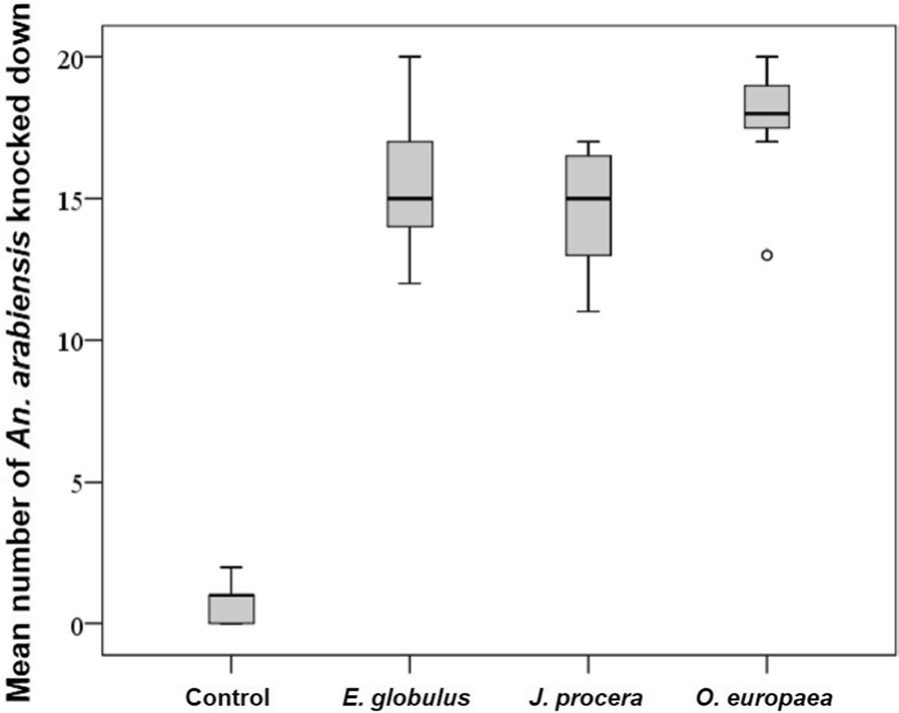
Mean number of *An. arabiensis* knocked down because of smoking of ethno-medicinal plants

Plants induced a high knockdown rate in *An. arabiensis* (*F* = 453; DF = 3; *P* < 0.001) compared to the control tents. The mean knockdown effect was relatively high due to *O. europaea* (17.7 ± 0.54; 95% CI 16.8–18.6), while it was only 0.9 ± 0.1; 95% CI 0.29–1.52 in control tents (Fig. 3).

### Feeding inhibition of smoking plants in the tent experiment

**Fig. 4:**
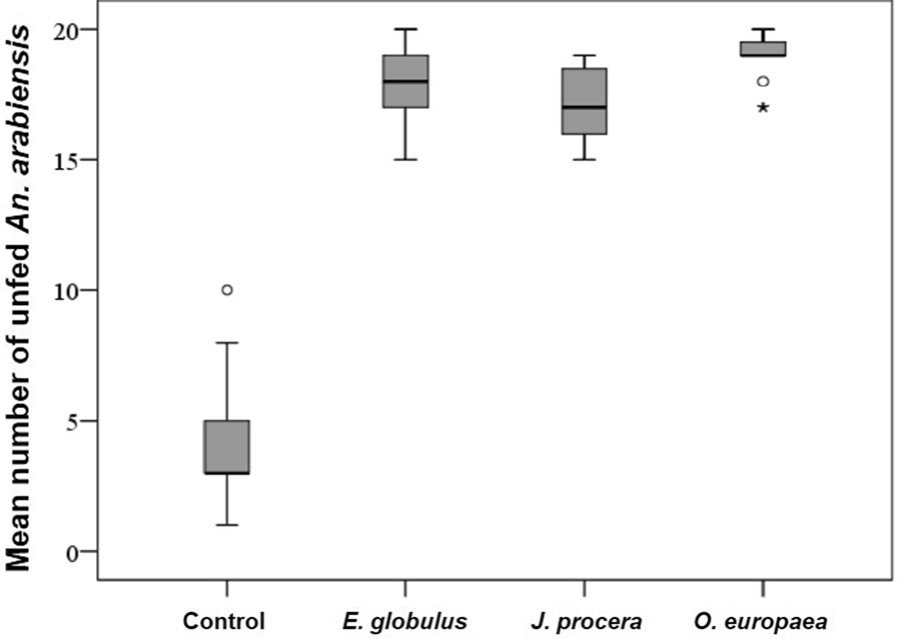
Mean number of unfed *An. arabiensis* in tents with smoking of ethno-medicinal plants and control tents

All the test plants used in the tent trial had significantly inhibited feeding activity of *An. arabiensis* (*F* = 383.5, DF = 3, *P *< 0.01). About 94.5% *An. Arabiensis* were unfed (with mean number of unfed 18.9 ± 0.24) in tents because of *O. europaea*, while only 19.5% were unfed in control tents (with mean 3.9 ± 0.42) (Fig. 4).

**Fig. 5:**
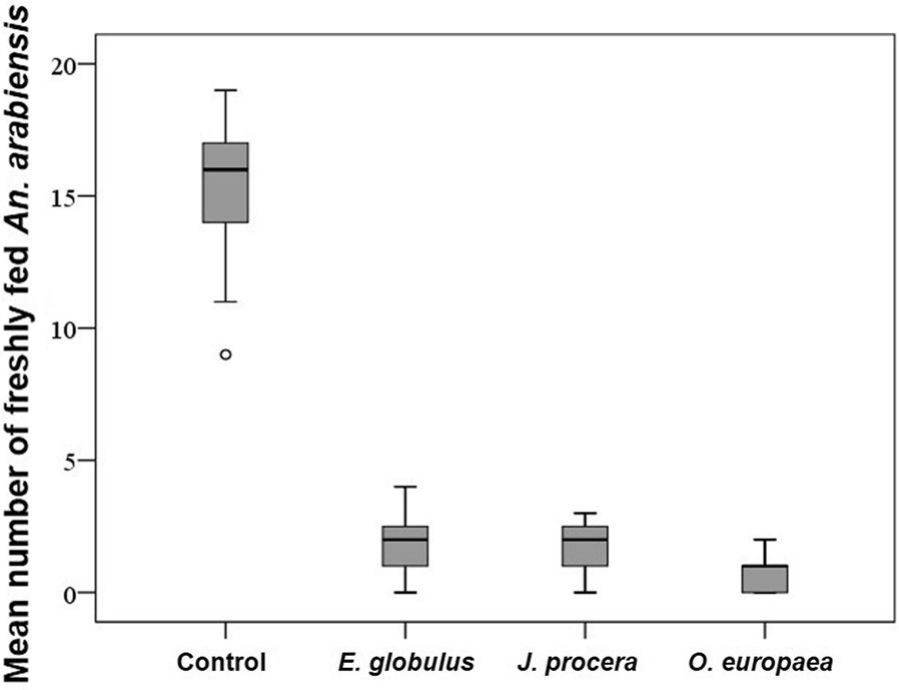
Mean number of freshly fed *An. arabiensis* released into control and treatment tents

The mean number (15.2 ± 0.5) of blood-engorged female *An. Arabiensis* was significantly higher in the control tent compared to the treatment groups. There was no significant difference in the proportion of blood-fed *An. arabiensis* according to the test plants (*P* < 0.01); smoking of *O. europaea, E. globulus* and *J. procera* inhibited *An. arabiensis* from feeding on the host (*F* = 344.8, DF = 3), and only a few *An. arabiensis* managed to feed on cattle in tents with smoking plants (Fig. 5).

## Discussion

The present study was carried out to evaluate the effect of smoking ethno-medicinal plants on indoor density and feeding activity of malaria vectors in Southern Ethiopia. The ethno-medicinal plants (*O. europaea, E. globulus* and *J. procera*) were tested by direct smoking with traditional clay stoves in the field and tents against host-seeking malaria vectors. The results of this study showed that there was a high repellent effect of ethno-medicinal plants on reducing indoor density of host-seeking malaria vectors under field evaluation. Moreover, the tent trial indicated smoking these plants inhibited feeding activity on cattle and had high knockdown effects of malaria vectors. The tested ethno-medicinal plants in both trial methods (tent and field) in the current study gave significant protection (> 60%) against the host-seeking malaria vectors in the study area.

The field trial revealed that the smoke emitted from burning ethno-medicinal plant-based material reduced the indoor density of host-seeking malaria vectors. The highest and least percentages of repellency against malaria vectors were observed for *O. europaea* and *E. globulus*, respectively. This percentage of protection was slightly higher than the reported repellent activity of aromatic plants by direct burning method against *An. arabiensis* and *An. pharoensis* in central Ethiopia [12]. The degree to which the plant materials were repellent varied according to the species of plant and the species of mosquito [17]. The finding from this study was in line with studies from different parts of Ethiopia [12, 18] and western Kenya [11] that indicated the ethno-medicinal plants materials had a significant repellent effect against malaria vectors.

According to the tent trial, smoke of *J. procera, E. globulus* and *O. europaea* inhibited the malaria vector from feeding on hosts and knocking down the vectors. This finding was comparable to similar studies that reported the knockdown as well as repelling effects of ethno-medicinal plant materials against host-seeking mosquitoes [14, 19]. The inhibition of feeding activity of malaria vectors due to smoking ethno-medicinal plants could be due to the volatile chemicals emitted from the plants. Smoke emitted from ethno-medicinal plants may interfere with chemo-receptors of mosquito by lowering the humidity since they respond more actively in the presence of moisture. For instance, *Eucalyptus* species contain eucalyptol, an active volatile substance, which repels host-seeking mosquitoes [17]. There are also reports about personal protection due to the repellency of *Eucalyptus* species against mosquitoes both outdoors and indoors [20] and long hour protection of *E. globulus* oil against the bites of *Ae. aegypti, An. stephensi* and *Cx. quinquefasciatus* [21]. In the present study, there were fewer blood-engorged mosquitoes in the tent with plant material smoking than plant smoke-free tents. This may be due to the volatiles involved in smoke-induced knockdown of the mosquitoes attempting to feed on animal hosts inside the tents. Moreover, the volatiles in smoke may block receptors and abolish upward flight and may also exhibit host recognition [11, 22]. Therefore, identifying the active volatile emitted in the form of smoke from plant materials could be a potential tool to repel malaria vectors and reduce biting on hosts.

*Juniperus procera, E. globulus* and *O. Europaea* are locally available and culturally acceptable plants in most rural communities of Ethiopia and are known for their ethno-medicinal values. They can be used as a promising local mosquito repellent to chase away mosquitoes by burning their leaves, fruit and bark [12, 19]. Furthermore, the method is simple and adaptable under varied natural situations in rural communities and hence could be integrated with existing tools. Over the past decades, there has been growing interest and hope related to designing potential plant-based insecticidal agents, which may have complex mechanisms of action to overcome insecticide resistance. In different parts of malaria-endemic countries, there is evidence that smoke produced by burning repellent ethno-medicinal plants is effective against malaria vectors and may be used as a supplementary malaria vector control strategy [17]. Therefore, ethno-medicinal plant-based mosquito control has potential to be used as a supplement to the key malaria control interventions (IRS and LLINs) by filling in their gaps. Thus, it may tackle residual malaria transmission by helping to avoid mosquito bites during the early hours of the night before bedtime.

As is the case in other studies, this study has its own strengths and limitations. As a strength, this study was an experimental trial which was conducted repeatedly in both the field and tent trials to ensure the impact of smoking ethno-medicinal plant materials. In addition, the study used CDC light traps for mosquito collection in the field trial, which is believed to minimize the bias due to collector skill. Nonetheless there are some limitations to the study. The bioactive molecules and their mode of action are not evaluated in this study. This was due to limited resources.

## Conclusions

In conclusion, smoke of local ethno-medicinal plants reduced the indoor density of host-seeking malaria vectors and inhibited feeding on hosts and induced knockdown in the tent experiment. Thus, smoking ethno-medicinal plants can be used as a supplementary vector control strategy to reduce indoor biting of mosquitoes, thereby limiting residual malaria transmission during the early hours of the night. Furthermore, it is recommended to isolate and identify bioactive molecules of test plants and their mode of action to prepare products that could be commercialized for vector control.

## Data Availability

Data supporting the conclusions and outcomes of this article are included in the article. The raw datasets presented and analyzed in this study are available upon request from the corresponding author.

## Abbreviations

CDC: Centers for Disease Control and Prevention
LLIN: Long-lasting insecticidal nets
IRS: Indoor residual spray
